# Gastrointestinal parasites of dromedary camel (*Camelus dromedarius*) in Algeria

**DOI:** 10.14202/vetworld.2020.1635-1640

**Published:** 2020-08-19

**Authors:** Messaoud Bouragba, AbdElkarim Laatamna, Fatima Elzahra Cheddad, Djamel Baroudi, Karim Houali, Ahcène Hakem

**Affiliations:** 1 Laboratory of Analytical Biochemistry and Biotechnology, Faculty of Biological and Agronomical Sciences, University of Mouloud Mammeri, Tizi-Ouzou, Algeria; 2 Laboratory of Exploration and Valorization of Steppic Ecosystems, Faculty of Nature and Life Sciences, University of Djelfa, Djelfa, Algeria; 3 Laboratory of Biomedical Analyses, Djelfa, Algeria; 4 Higher National Veterinary School, Issad Abbes Street, Algiers, Algeria; 5 Centre Research in Agro-Pastoralism, Djelfa, Algeria

**Keywords:** Algeria, camel dromedary, helminths, prevalence, protozoans

## Abstract

**Aim:**

The present study was designed to investigate the prevalence and identification of gastrointestinal parasites in feces samples of dromedary camels (*Camelus dromedarius*) in Algeria based on microscopic examination.

**Materials and Methods:**

A total of 717 fresh fecal samples obtained from 28 farms at Steppe and Northern Sahara regions of Algeria were processed for microscopic examination after concentration by formalin-ether sedimentation and flotation techniques. In addition, microscopic examination of *Cryptosporidium* spp. was done by modified Ziehl-Neelsen staining and Lugol staining procedure was used for the detection of Giardia cysts.

**Results:**

Microscopic examination indicated an infection rate of gastrointestinal parasites of 48.26% (346/717). Protozoan infections were recorded at 17.02% (122/717), whereas helminth infections were recorded at 23.71% (170/717). In addition, mixed infection (protozoans and helminths) was seen at 7.53% (54/717). No correlation was found between infection and age of the animals, nor the consistency of the stool samples; in addition, neither influence of sex nor breed of camels was observed. Eighteen genera of gastrointestinal parasites were revealed; including four genera of protozoa, 12 Nematoda, one Cestoda, and one Trematoda. *Strongyloides* spp. and *Eimeria* spp. showed the highest rate of parasitism, while *Cooperia* spp. was observed with the lowest prevalence. *Cryptosporidium* spp. was detected in 13 among 717 examined samples (1.81%).

**Conclusion:**

The parasite fauna infecting the gastrointestinal tract of the Algerian dromedary is much diversified. The detected parasites in camels are similar to counterparts in other ruminants, posing serious challenge to animal farming. Future studies should be carried out to better understand the epidemiology of these parasitic diseases and their economic and public health impact.

## Introduction

Historically, camels are important animals for their meat and milk production as well as transportation, especially across deserts in many African and Asian countries. However, they are prone to infection by many parasitic diseases [1-5] resulting in considerable economic losses related to decrease in productivity and performance as well as mortality in severe cases [5,6].

Gastrointestinal parasites, including protozoa and helminths, are common findings in camel populations in different countries [2,7-10]. In Algeria, the camel farming sector (354.465 camel heads) has a substantial contribution to cover the growing gap in protein and dairy products. However, camel farming suffers from many debilitating diseases, including vector-borne and blood infections such as babesiosis, rickettsiosis, and trypanosomosis [11-13] as well as tissue dwelling parasites such as *Echinococcus* spp. [14,15]. Notwithstanding, little is available on the gastrointestinal parasitic infections in dromedary camels in Algeria [16,17].

The present study was designed to investigate the prevalence and identification of gastrointestinal parasites in camels in Algeria based on the microscopic examination. To this end, fecal samples collected from dromedary camels at Steppe and Northern Sahara regions of Algeria were investigated using the coproscopic examination.

## Materials and Methods

### Ethical approval and informed consent

The study protocol was approved by the Laboratory of Exploration and Valorization of Steppic Ecosystems, Faculty of Nature and Life Sciences, University of Djelfa. Informed written consent was obtained from farm owners or managers. Camels were handled in compliance with the established regulations and guidelines in Algeria.

### Study area

**Figure-1:**
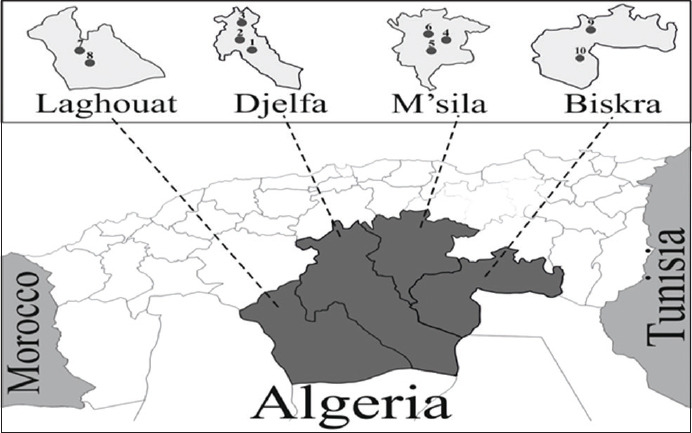
Map of Algeria showing the locations of examined herds of dromedary camel in the steppe (Djelfa, M’sila) and Northern Saharan (Laghouat, Biskra) regions.

This study was carried out between March 2015 and July 2018 on 28 camel herds in M’sila and Djelfa provinces at the steppe region in the central part of Northern Algeria; as well as Biskra and Laghouat in the Northern part of the Algerian Sahara (Figure-1). Djelfa and M’sila regions are generally dominated by a semi-arid climate (low and irregular precipitation) with an average annual temperature during the study period of 15.7 °C and 19.2 °C respectively. While Biskra and Laghouat are dominated by arid climate with an average annual temperature of 23.3 °C and 19.8 °C, respectively. One feature worth mentioning, the northwestern part of the Laghouat region is characterized during the winter by snowfall and strong periods of frost. Camels graze on pasture during most of the year. The study area (either the steppe region or Northern part of the Algerian Sahara has extensive small ruminant (sheep and goat) farming activity.

### Sample collection

A total of 717 fecal samples were obtained from camels either directly from their rectum or from the ground immediately after defecation. Each sample was individually placed into a sterile plastic container, labeled with the epidemiological data, and transported in an isotherm box to the laboratory.

### Coprological examination

The samples were subjected to macroscopic examination to assess the consistency of the feces and to check for visible parasitic elements. Fecal samples were processed for microscopic examination after concentration by formalin-ether sedimentation [18]. In addition, all samples were processed by floatation technique (NaCl solution of 1.2 density). The microscopic examination of *Cryptosporidium* spp. was done by modified Ziehl-Neelsen staining procedure [19] and Lugol staining procedure was used for the detection of *Giardia* cysts. Genus identification was carried out based on the detection of eggs and oocysts excretion in the feces; no adult or larval stages (for helminths) were used to determine the type of parasite.

To study the degree of infestation of *Cryptosporidium* spp. oocysts, a scoring system for positive specimens can be used based on a semi-quantitative technique. Under the ×40 objective, the number of oocysts was calculated in 200 fields per slide on average. However, this technique cannot be considered an accurate quantitative measurement because the number of oocysts changes considerably during the course of infection. The score for each positive slide was established as follows: (+) less than 5 oocysts per slide, (++) 1 to 10 oocysts per field of view, and (+++) 11 or more oocysts per field of view [20].

### Statistical analysis

Excel software was used to perform the statistical analysis. The Chi-square test was exploited to assess relationships between the parasitism rate and animal attributes (age, sex, breed, and presence or absence of diarrhea). The confidence interval was fixed at 95% and the Chi-square value was calculated with p<0.05 which was considered as statistically significant.

## Results

The microscopic examination revealed an overall infection rate of 48.26% (346/717). Infected camels were found to be harboring eggs or oocysts at least one genus of gastrointestinal parasites. The parasitic infection was recorded in all examined farms (Table-1).

**Table-1:** Overall infection rate in dromedary farms sampled from the Steppe and Northern Saharan regions.

Province	Locality	Sampled herds	Examined camels	Overall infection		*Cryptosporidium* spp.	
	
				n	Prevalence (%)	n	Prevalence (%)
Djelfa	Musrane^1^	7	188	86	45.74	5	2.66
	Zaafrane^2^	3	55	31	56.36	0	0
	Boughezoul^3^	6	165	93	56.36	4	2.42
M’sila	Maarif^4^	4	87	44	50.57	3	2.91
	Zerarga^5^	1	19	1	5.26	0	0
	Bainou^6^	1	16	12	75.0	0	0
Laghouat	Tadjmout^7^	1	15	5	33.33	0	0
	Berkane^8^	1	56	26	46.43	0	0
Biskra	Toulga^9^	3	103	40	38.83	1	0.97
	Sidi Khaled^10^	1	13	8	61.54	0	0
Total	10	28	717	346	48.26	13	1.81

^1-10^Correspond to the localities shown in Figure-1

From the 346 positive cases, 239 (33.33%) were those with single infection, i.e., each animal was infected only by one parasite type. The other infections were multiple (either double, triple, or quadruple with infection cases of 82, 21, and 3, respectively). In addition, one 9-month-old camel was found to be infected by five genera of parasites.

Protozoan infections were observed in 122/717 cases (17.02%), whereas helminth infections were observed in 170/717 (17.02%) camels. In addition, mixed infections of protozoan and helminth were recorded in 54/711 (7.53%) animals. Occurrence of infection was similar in female and male camels (242/508; 47.64%) for the former, and (104/209; 49.76%) for the latter. Furthermore, no correlation was found between infection and age of the animals, nor the consistency of the stool samples (presence or absence of diarrhea). Neither breed of the camels nor area had influence on the infection rate (Table-2).

**Table-2:** Distribution of the parasitism rate by sex, age, presence or absence of diarrhea, breed, and area.

Variable	Examined camels	Overall infection		*Cryptosporidium* spp.	
	
		n	Prevalence (%)	n	Prevalence (%)
Gender					
Male	209	104	49.76	3	1.44
Female	508	242	47.64	10	1.97
χ^2^ (p-value)		0.267 (0.605)		0.236 (0.627)	
Age					
<1 years	279	138	49.46	3	1.08
1-4 years	181	92	50.83	9	4.97
4-9 years	182	81	44.51	1	0.55
>9 years	75	35	46.67	0	0
χ^2^ (p-value)		1.744 (0.627)		14.02 (0.009)	
Diarrhea					
Presence	20	9	45.00	1	5.00
Absence	697	337	48.35	12	1.72
χ^2^ (p-value)		0.087 (0.768)		1.174 (0.279)	
Breed					
Ouled-Nail	314	149	47.45	5	1.59
Sidi-Cheik	122	56	45.90	3	2.46
Chaambi	204	102	50.00	5	2.45
Reguibi	77	39	50.65	0	0
χ^2^ (p-value)		0.739 (0.864)		2.260 (0.520)	
Area					
Steppe (Semi-arid)	530	267	50.38	12	2.26
North. Sahara (Arid)	187	79	42.25	1	0.53
χ^2^ (p-value)		3.660 (0.056)		2.322 (0.128)	
Total	717	346	48.26	13	1.81

*Cryptosporidium* spp. was detected in four localities (Table-1) with an overall prevalence of 1.81% (13/717). *Cryptosporidium* excretion did not vary significantly by sex, nor breed, or origin region. Out of 20 diarrheic camels, only one animal showed *Cryptosporidium* spp. excretion. Therefore, this presence was not significantly associated with diarrhea. However, a significant difference (p=0.009) was observed between the presence of *Cryptosporidium* spp. and age of examined camels. The higher rate of infection (9/181; 4.97%) was recorded in animals aged between 1 and 4 years, while no infection was recorded for animals older than 9 years (Table-2). Among infected camels (13 cases), nine positive cases showed *Cryptosporidium* excretion with a mean score (<5 oocysts/slide). Meanwhile, two infected camels showed a middle score (1-10 oocysts/field of view). There were no positive cases where the number of oocysts is more than 11 oocysts per field of view (Table-3).

**Table-3:** Degree of infestation by *Cryptosporidium spp.* oocysts in dromedary camel.

	Total	Degree of infestation		

		1-5 oocysts (+)	1-10 oocysts (++)	>10 oocysts (+++)
Number of infected camels	13	11	2	0

The results showed that many parasitic classes infect dromedary camel. The highest rate was 21.76% (156/717 were infested with Nematoda parasites); Trematoda was the lowest at 0.28% (Table-4). In total, 18 genera of gastrointestinal parasites were recorded in the present investigation; including four genera of protozoa and 14 ones of helminthes (12 *Nematoda*, one *Cestoda* and one *Trematoda*). *Strongyloides* spp. (11.16%) and *Eimeria* genus (10.32%; data not shown) showed the highest rate of parasitism. In addition, *Eimeria* oocysts were identified as *Eimeria cameli* (14 cases; 1.95%), *Eimeria dromedarii* (37 cases; 5.16%), and there main as *Eimeria* spp. (23 cases; 3.21%), while, *Cooperia* spp. was lowest in prevalence with two cases (0.28%) (Table-5).

**Table-4:** Infection rate of identified categories gastrointestinal parasites in examined camels.

Identified parasites	Number of infested camels	Prevalence (%)
Protozoa	122	17.02
Nematoda	156	21.76
Cestoda	12	1.67
Trematoda	2	0.28
Mixed infection	54	7.53
Total	346	48.26

**Table-5:** Infection rate of identified genera of gastrointestinal parasites in examined camels.

Identified parasites	Number of infested camels	Prevalence (%)
Protozoa		
*Eimeria* spp.	23	3.21
*Eimeria cameli*	14	1.95
*Eimeria dromedarii*	37	5.16
*Neobalantidium* spp.	54	7.53
*Buxtonella* spp.	50	6.97
*Cryptosporidium* spp.	13	1.81
Helminths		
*Strongylus* spp.	31	4.32
*Marshallagia* spp.	59	8.23
*Nematodirus* spp.	38	5.30
*Trichuris* spp.	16	2.23
*Bunostomum-like* spp.	6	0.84
*Strongyloides* spp.	80	11.16
*Oesophagostomum* spp.	4	0.56
*Trichostrongylus* spp.	4	0.56
*Dictyocaulus* spp.	4	0.56
*Chabertia* spp.	3	0.42
*Ostertagia* spp.	3	0.42
*Cooperia* spp.	2	0.28
*Moniezia* spp.	31	4.32
*Fasciola* spp.	3	0.42

## Discussion

Little is known about epidemiology of gastrointestinal parasitic infections in dromedary camels in Algeria. The results of the present study indicated that the overall infection rate was 48.26% (346/717) of investigated camels at Steppe and Northern Sahara regions of Algeria. These findings are in agreement with those reported previously from different African and Asian countries [6,21-23]. It is worth mentioning that parasitic infections of camels are likely to depend on environmental and host-related factors. Climatic conditions and husbandry practices are important environmental factors, whereas, animal breed, age, and population density are host-related factors. Furthermore, investigation method, sampling time, and number of screened animals are important ones. These factors may have individually or collectively contributed to the high infection rate recorded in the present study.

The results of the present study indicated that helminthic infections were (170/717; 23.71%) compared to protozoan infections (122/717; 17.02%) in the investigated camels. These findings are similar to those reported in the previous studies [22,24]. It is evident that the helminthic and protozoan infections are commonly associated with livestock, including camels.

No statistical differences were found between different age categories and excretion of endoparasites detective stage, contrasting results reported by Bekele [6] state that parasite excretion was more recorded in adult animals compared to young ones. In concordance with Radfar and Aminzadeh [22], no significant difference was recorded in parasitic excretion and the sex of the camel. Contrariwise, it has been reported that the females were more prone to parasitic infection than males [6,21]. Noteworthy, the breed of camels had no influence on the occurrence of infection, the implication of this factor in the excretion of internal parasites is poorly known, and so further studies are needed to clarify this situation.

Despite the low number of investigated diarrheic camels (20/717) in the present survey, the infection rate in these camels (9/20) did not differ from that in non-diarrheic ones. Although it is difficult to explain the causes of diarrhea in camels, nutritional factors and/or gastrointestinal infections are important elements. Furthermore, the load of infectious parasites play a crucial role in instigating diarrhea [5,25].

The results of the present study identified diverse genera of nematodes and protozoans recorded in investigated camels (Table-5), with *Strongyloides* spp. and *Eimeria* spp. were the most prevalent parasites, which was not the case in several studies [21-23]. Meanwhile, *Oesophagostomum* spp., *Trichostrongylus* spp., *Dictyocaulus* spp., *Chabertia* spp., *Ostertagia* spp., *Cooperia* spp., and *Fasciola* spp. were observed with the lower prevalence. In general, the findings of the present study regarding the infection rate of each detected genre are lower compared to those reported previously from different African and Asian countries.

It is worth noting that the prevalence of cryptosporidiosis in Algerian dromedary camel has been scarcely dealt with. Only two previous Algerian studies reported a prevalence of 5.13% [16] and 2.01% [17]. The prevalence of the present study (1.81%) is slightly lower than those reported in Algeria and even in Egypt (3.4%) [26]. However, our prevalence is similar to that from Iran (1.9%) [7], whereas, it is significantly very low when compared to results from other some previous studies conducted in Iran (37.9%) and in Iraq (61%) [27,28].

The age group of camels has a significant effect on the presence of cryptosporidiosis infection that is observed in the present report as well as conducted in several other studies [26,28,29]. Notwithstanding, no statistical difference was found between the age of camels and the presence of *Cryptosporidium* spp. in some Iranian reports [5,27]. The area of origin (steppe and Sahara), sex, breed, and diarrheal status was not reported as an associated risk factors in the prevalence variation of *Cryptosporidium* spp. A little is known concerning the zoonotic potential of dromedary camel cryptosporidiosis. *C. parvum*, *C. andersoni*, *Cryptosporidium* rat genotype IV, and a novel genotype (named “camel genotype”) are known to infect dromedary camel [30]. Only *C. parvum* (subtype IIaA17G2R1) that detected in dromedary camel represents a common zoonotic subtype reported in humans and animals worldwide [31]. In addition to *Cryptosporidium parvum*, dromedary camel can play a role as potential reservoir of major zoonotic parasites transmitted to humans through direct/indirect contamination such as *Giardia duodenalis*, *Blastocystis* spp. and *Enterocytozoon bieneusi*, as food-borne infections such as *Toxoplasma gondii* and *Trichinella* spp., and by arthropod vectors including *Trypanosoma* spp [30]. Some parasites common to ruminants and camels like *Trichostrongylus* have minor public health significance and can occasionally infect human. Among more than 30 species within *Trichostrongylus* genus, ten different species have been reported in humans including *T. colubriformis* that representing the main zoonotic species [32,33]. The morphology of *Cryptosporidium* oocysts and *Trichostrongylus* eggs does not allow anyway the differentiation between the different species within these two parasite genus. Therefore, in the present study, molecular analysis is required for species identification and highlighting of their zoonotic potential.

## Conclusion

The parasite fauna infecting the gastrointestinal tract of the Algerian dromedary is much diversified, consisting of different species of helminths and protozoa, common to other ruminants. They can affect the growth and productivity of animals and cause clinical signs of diarrhea as well. Future studies should be carried out to better understand the epidemiology of these parasitic diseases and investigate their economic and public health impact.

## Authors’ Contributions

MB, FEC, and AKL collected the samples and did the laboratory analysis. MB carried out the statistical analysis and data curation. AH and KH designed the study and laboratory work. AKL and MB wrote the manuscript. DB and AH helped in the writing and review. All authors have read and approved the final manuscript.

## Competing Interests

The authors declare that they have no competing interests.

## Publisher’s Note

Veterinary World remains neutral with regard to jurisdictional claims in published map and institutional affiliation.
